# Complete Genome Sequence of a Highly Virulent Newcastle Disease Virus Currently Circulating in Mexico

**DOI:** 10.1128/genomeA.00177-12

**Published:** 2013-02-14

**Authors:** Sa Xiao, Anandan Paldurai, Baibaswata Nayak, Armando Mirande, Peter L. Collins, Siba K. Samal

**Affiliations:** a Virginia-Maryland Regional College of Veterinary Medicine, University of Maryland, College Park, Maryland, USA; b Supervet, Inc., The Woodlands, Texas, USA; c Laboratory of Infectious Diseases, National Institute of Allergy and Infectious Diseases, National Institutes of Health, Bethesda, Maryland, USA

## Abstract

The complete genome sequence was determined for a highly virulent Newcastle disease virus strain from vaccinated chicken farms in Mexico during outbreaks in 2010. On the basis of phylogenetic analysis this strain was classified into genotype V in the class II cluster that was closely related to Mexican strains that appeared in 2004–2006.

## GENOME ANNOUNCEMENT

Newcastle disease (ND) is a highly contagious viral disease in avian species and causes large economic losses in the poultry industry. Newcastle disease virus (NDV) is a member of the genus *Avulavirus* in the family *Paramyxoviridae*. The genome of NDV is a single-stranded, negative-sense, nonsegmented RNA with six genes encoding seven major viral proteins (1). NDV strains are divided into two classes based on genetic analysis: class I strains have been isolated mainly from wild birds and are generally avirulent, whereas class II strains have been recovered from wild and domestic birds and include virulent and avirulent isolates (2). Class I and II viruses are divided into 9 and 11 genotypes, respectively (3). Currently circulating strains in chickens around the world are genotypes V, VI, and VII of class II. Although all commercial chickens are routinely vaccinated with live NDV vaccine B1 or LaSota, ND continues to be a major problem for the poultry industry (4).

In North America, Mexico is the only country where highly virulent NDV strains are circulating (4). In 2010, NDV outbreaks affected chicken farms located in the states of Coahuila and Durango in North Central Mexico, in an agricultural region known as La Laguna in Mexico. ND outbreaks occurred in commercial vaccinated chickens, causing up to 24% to 36% mortality. Tissue samples, including tissue from lungs, trachea, spleen, and cecal tonsils, were taken from dead and sick broilers exhibiting central nervous system (CNS) signs and severe dyspnea. One of the isolates, namely, Mexico/01/10, was purified, and the genome sequence was determined by reverse transcription (RT)-PCR using overlapped consensus primers and direct sequencing. The 3′ and 5′ termini were determined by rapid amplification of cDNA ends (RACE) (5). The complete genome of Mexico/01/10 is 15,192 nucleotides in length. The amino acid sequence identities of fusion (F) and hemagglutinin-neuraminidase (HN) proteins between Mexico/01/10 and Mexican strains isolated in 2004–2006 are 99% and 98%, respectively (6, 7). However, the amino acid sequence identity of the F and HN proteins between Mexico/01/10 and the currently used vaccine strain LaSota is 89%. This indicates that the circulating strains are substantially distinct from the vaccine strain in use and suggests that antigenic differences contributed to poor vaccine protection.

The sequence of the F protein cleavage site is a major determinant of NDV pathogenicity. The cleavage sites of virulent NDV strains usually contain multiple basic residues, whereas avirulent strains have fewer basic residues (8, 9). The Mexican isolate has a virulent pathotype: RRQKR↓F (underlining represents the basic residue; arrow represents the site of cleavage). Phylogenetic analysis of the complete coding region of the F gene constructed by MEGA4.0 indicated that it was within genotype V in class II and is more closely related to Mexico isolates that appeared in 2004–2006, demonstrating that the highly virulent NDV strains are still circulating in Mexico with minor genetic variations.

### Nucleotide sequence accession number.

The complete genome sequence of Mexico/01/10 has been deposited in GenBank under the accession number JX974435.
